# Short‐term safety of an anti‐severe acute respiratory syndrome coronavirus 2 messenger RNA vaccine for patients with advanced lung cancer treated with anticancer drugs: A multicenter, prospective, observational study

**DOI:** 10.1111/1759-7714.14281

**Published:** 2021-12-28

**Authors:** Tomoki Tamura, Kiichiro Ninomiya, Toshio Kubo, Shoichi Kuyama, Sayaka Tachibana, Koji Inoue, Kenichi Chikamori, Kenichiro Kudo, Nobuaki Ochi, Daijiro Harada, Yoshinobu Maeda, Katsuyuki Kiura

**Affiliations:** ^1^ Department of Respiratory Medicine NHO Iwakuni Clinical Center Iwakuni Japan; ^2^ Department of Hematology, Oncology and Respiratory Medicine Okayama University Graduate School of Medicine, Dentistry and Pharmaceutical Sciences Okayama Japan; ^3^ Center for Clinical Oncology Okayama University Graduate School of Medicine, Dentistry and Pharmaceutical Sciences Okayama Japan; ^4^ Department of Respiratory Medicine Ehime Prefectural Central Hospital Matsuyama Japan; ^5^ Department of Medical Oncology National Hospital Organization Yamaguchi‐Ube Medical Center Ube Japan; ^6^ Department of Respiratory Medicine National Hospital Organization Okayama Medical Center Okayama Japan; ^7^ General Internal Medicine 4 Kawasaki Medical School Okayama Japan; ^8^ Department of Thoracic Oncology NHO Shikoku Cancer Center Matsuyama Japan; ^9^ Department of Allergy and Respiratory Medicine Okayama University Hospital Okayama Japan

**Keywords:** anticancer drugs, COVID‐19, lung cancer, SARS‐CoV‐2, vaccine

## Abstract

**Background:**

Since 2020, severe acute respiratory syndrome coronavirus 2 (SARS‐CoV‐2) has become prevalent worldwide. In severe cases, the case fatality rate is high, and vaccine prevention is important. This study evaluated the safety of receiving SARS‐CoV‐2 vaccine in patients with advanced lung cancer receiving anticancer therapy.

**Methods:**

We prospectively enrolled patients receiving anticancer drugs for advanced lung cancer who planned to receive SARS‐CoV‐2 vaccination. Early adverse events within 7 days of vaccine injection were evaluated using patient‐reported surveys. The chi‐square test and multivariate logistic regression analyses were used.

**Results:**

Among 120 patients receiving lung cancer treatment, 73 were men; the mean age of the patients was 73.5 years. The treatments received for lung cancer at the time of the first vaccine injection were chemotherapy, ICIs, combined chemotherapy and ICIs, and targeted therapies, including tyrosine kinase inhibitors, in 30, 28, 17, and 45 patients, respectively. All patients received SARS‐CoV‐2 messenger RNA (mRNA) vaccine. After the second mRNA vaccine dose, 15.4% of patients had fever of 38°C (95% confidence interval: 9.34%–23.2%); this rate was slightly higher than that for healthy participants at the time of the BNT162b2 trial. Patients treated with cytotoxic anticancer drugs tended to have high fever. In the multivariate analyses, male sex was associated with higher fever frequencies. However, there were no serious early adverse events due to vaccination.

**Conclusions:**

Anti‐SARS‐CoV‐2 mRNA vaccination tends to be safe, but fever following vaccination tends to be more common among patients undergoing lung cancer treatment than among healthy individuals.

## Graphical abstract

- Anti‐SARS‐CoV‐2 messenger RNA vaccination itself tends to be safe.
- Vaccine‐related AEs tend to increase in lung cancer patients with chemotherapy.
- Serious adverse events are comparable to those in healthy individuals.

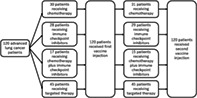

## INTRODUCTION

Since 2020, there have been outbreaks of severe acute respiratory syndrome coronavirus 2 (SARS‐CoV‐2) infection worldwide. SARS‐CoV‐2 infection is severe in patients with lung cancer.^1^, ^2^, ^3^, ^4^ Steroids, antiviral drugs, anti‐interleukin‐6 drugs, etc. are currently used for treating SARS‐CoV‐2. However, there has been no silver bullet breakthrough as yet. The effectiveness of treatment that prevents aggravation, such as antibody cocktail therapy^5^ and new antiviral drugs,^6^ has been reported. The usefulness of the messenger RNA (mRNA) vaccine has already been reported^7^, ^8^ and BNT162b2 and mRNA‐1273 are typical SARS‐CoV‐2 mRNA vaccines currently is used worldwide. Vaccination is recommended for patients with cancer by the Center for Disease Control and Prevention and National Comprehensive Cancer Network.^9^, ^10^ However, few data are available to establish vaccine safety and efficacy in patients with advanced cancer. The SARS‐CoV‐2 vaccine has a high incidence of side effects such as fever even in healthy individuals, but there are few serious adverse events. In the BNT162b2 phase 3 trial, only approximately 4% of enrolled patients had a malignancy of any type, and these patients were not analyzed separately to assess vaccine efficacy.^7^ In the mRNA‐1273 trial, patients with cancer were not enrolled.^8^ There are reports that the antibody titer after vaccination does not change between patients with cancer and healthy people,^11^, ^12^, ^13^ while others have reported that antibody titers after mRNA vaccination are low in patients with solid cancer receiving anticancer drug treatment.^14^, ^15^ The SARS‐CoV‐2 vaccine has a high incidence of side effects such as fever even in healthy individuals. However, there are few serious adverse events.^7^, ^8^ The side effects of the vaccine in patients with chronic inflammatory diseases who are receiving immunosuppressive treatment are the same as those of healthy individuals.^16^ Although some reports have been published on the safety of SARS‐CoV‐2 mRNA vaccines in patients with cancer and it has been reported that there is no problem with safety,^17^ there are also case reports of cytokine release syndrome.^18^

Therefore, this study aimed to evaluate the safety of the vaccine in patients with lung cancer receiving anticancer drug therapy.

## METHODS

### Ethics statements

All participants provided written informed consent. This study was approved by the relevant institutional review board (National Hospital Organization Iwakuni Clinical Center Institutional Review Board, Iwakuni, Yamaguchi, Japan) (no. 0262) and was conducted in compliance with the Declaration of Helsinki and Ethical Guidelines for Medical and Health Research Involving Human Subjects. The study protocol was registered on the website of the University Hospital Medical Information Network, Japan (protocol ID: UMIN000043918).

### Study design and participants

This multicenter prospective observational study, the OLCSG2102 study, included patients with advanced lung cancer who were receiving anticancer therapies such as chemotherapy, immune checkpoint inhibitors (ICIs), and molecular targeted therapy. Patients who met the following eligibility criteria were enrolled at seven hospitals in Japan. They included those aged 20 years or older, diagnosed with unresectable cancer or recurrent lung cancer, receiving anticancer drug therapy, and scheduled for SARS‐CoV‐2 vaccination. Patients with a history of coronavirus disease (COVID‐19), patients with a history of SARS‐CoV‐2 vaccination, patients who were considered inappropriate for SARS‐CoV‐2 vaccination, or patients with an estimated prognosis of <2 months were excluded.

### Outcomes

The coprimary outcomes were to assess the frequency of fever and other side reactions 7 days after the second dose of SARS‐CoV‐2 vaccination based on a patient‐reported survey,

secondary outcomes of the frequency of fever and other side reactions 7 days after the first dose of SARS‐CoV‐2 vaccination based on the patient‐reported survey, incidence of grade 3 or worse immune‐related adverse events after SARS‐CoV‐2 vaccination in patients receiving ICIs, incidence of COVID‐19 after vaccination, overall survival after vaccination, and progression‐free survival of anticancer drug therapy. Body temperature of the axilla was measured in degrees Celsius.

### Data collection

The side reaction rating scale for the vaccine was based on the BNT162b2 report.^7^ Data on local and systematic reactions and use of medication were collected from patients who had been surveyed for 7 days after each vaccination. Pain at the injection site was assessed according to the following scale: mild, does not interfere with activity; moderate, interferes with activity; severe, prevents daily activity; and grade 4, emergency department visit or hospitalization. Redness and swelling were measured according to the following scale: mild, 2.0–5.0 cm in diameter; moderate, >5.0–10.0 cm in diameter; severe, >10.0 cm in diameter; and grade 4, necrosis or exfoliative dermatitis (for redness) and necrosis (for swelling). The scales of systematic events were as follows: fatigue, headache, chills, muscle pain, joint pain (mild, does not interfere with activity; moderate, some interference with activity; or severe, prevents daily activity), vomiting (mild, 1–2 times in 24 h; moderate, >2 times in 24 h; or severe, requires intravenous hydration), and diarrhea (mild, 2–3 loose stools in 24 h; moderate, 4–5 loose stools in 24 h; or severe, ≥6 loose stools in 24 h). Grade 4 for all events indicated an emergency department visit or hospitalization. Vaccine adverse reactions were reported daily by the patients on a predistributed questionnaire. Patients measured and recorded body temperature daily for 8 days from the day before vaccination to the seventh day after vaccination.

### Statistical analyses

Data from the BNT162b2 clinical trial showed that the frequency of fever >38°C after the second vaccination was 11% in the setting of patients aged 56 years or older.^7^ We assumed that a 10% increase in the frequency of fever >38°C in patients who were undergoing treatment for lung cancer would be acceptable. Accordingly, we estimated that the required number of patients for the early safety assessment would be 104, with a one‐sided significance level of 0.05 and a power of 80%. An interim analysis was conducted when 120 cases were collected considering that 5% of cases would drop out of the survey.

Differences were assessed using analysis of variance or chi‐square test. Adjusted odds ratios were calculated using multivariate logistic regression analyses with the following covariates: sex, age, smoking history, the presence of respiratory complications, and type of treatment. All statistical analyses were performed using a standard software package (STATA version 17; StataCorp). The significance threshold was set at *p* < 0.05 for the two‐sided unpaired tests.

## RESULTS

We report the results of the interim analysis of early adverse events owing to vaccination. Between April 8, 2021 and August 31, 2021, >400 patients undergoing lung cancer treatment were enrolled to assess vaccination safety and immune‐related adverse events. At the time of obtaining the patient‐reported survey from 120 patients to assess post‐vaccination safety, the initial adverse events of vaccination were analyzed. All patients received two doses of the vaccination.

Patient characteristics of the initial 120 patients are presented in Table 1. The patients comprised 73 men (61%) and 47 women (39%). The median age was 73.5 years (range, 64–86 years), and 41% of patients were 75 years or older. There were 74 (62%) smokers. All patients had advanced lung cancer, and the histological subtypes were mostly adenocarcinoma (*n* = 94). The treatments received for lung cancer at the time of the first vaccine injection were chemotherapy in 30 patients, ICIs in 28 patients, combination of chemotherapy and ICIs in 17 patients, and targeted therapies such as tyrosine kinase inhibitors in 45 patients. Two patients changed their treatment regimens between the first and second injections. Both patients were treated with a combination of chemotherapy and ICIs, and one patient's treatment was changed to chemotherapy alone and the other to ICI alone. In this study, 115 of the 120 patients received the BNT162b2 vaccine (Table 1).

**TABLE 1:** Patient characteristics

Characteristic	No.	%
Total	120	100
Age (years)		
Median (range)	73.5 (64–86)	
Sex		
Male/female	73/47	61/39
ECOG‐PS		
0/1/2	43/75/2	36/63/2
Smoking status		
Never smoked/smokers	46/74	38/62
Histology		
Ad/Sq/NOS/SCLC	94/15/3/8	78/13/3/7
Vaccine		
BNT162b2/mRNA‐1273/unknown	115/1/4	96/1/3
Treatment (first vaccine injection)		
Chemotherapy	30	25
ICI	28	23
Chemotherapy plus ICI	17	14
Targeted therapy	45	38
Treatment (second vaccine injection)		
Chemotherapy	31	26
ICI	29	24
Chemotherapy plus ICI	15	13
Targeted therapy	45	38

Abbreviations: Ad, adenocarcinoma; ECOG‐PS, Eastern Cooperative Oncology Group Performance Status; ICI, immune checkpoint inhibitor; mRNA, messenger RNA; no., number; NOS, not otherwise specified; SCLC, small cell lung cancer; Sq, squamous cell carcinoma.

**FIGURE 1:**
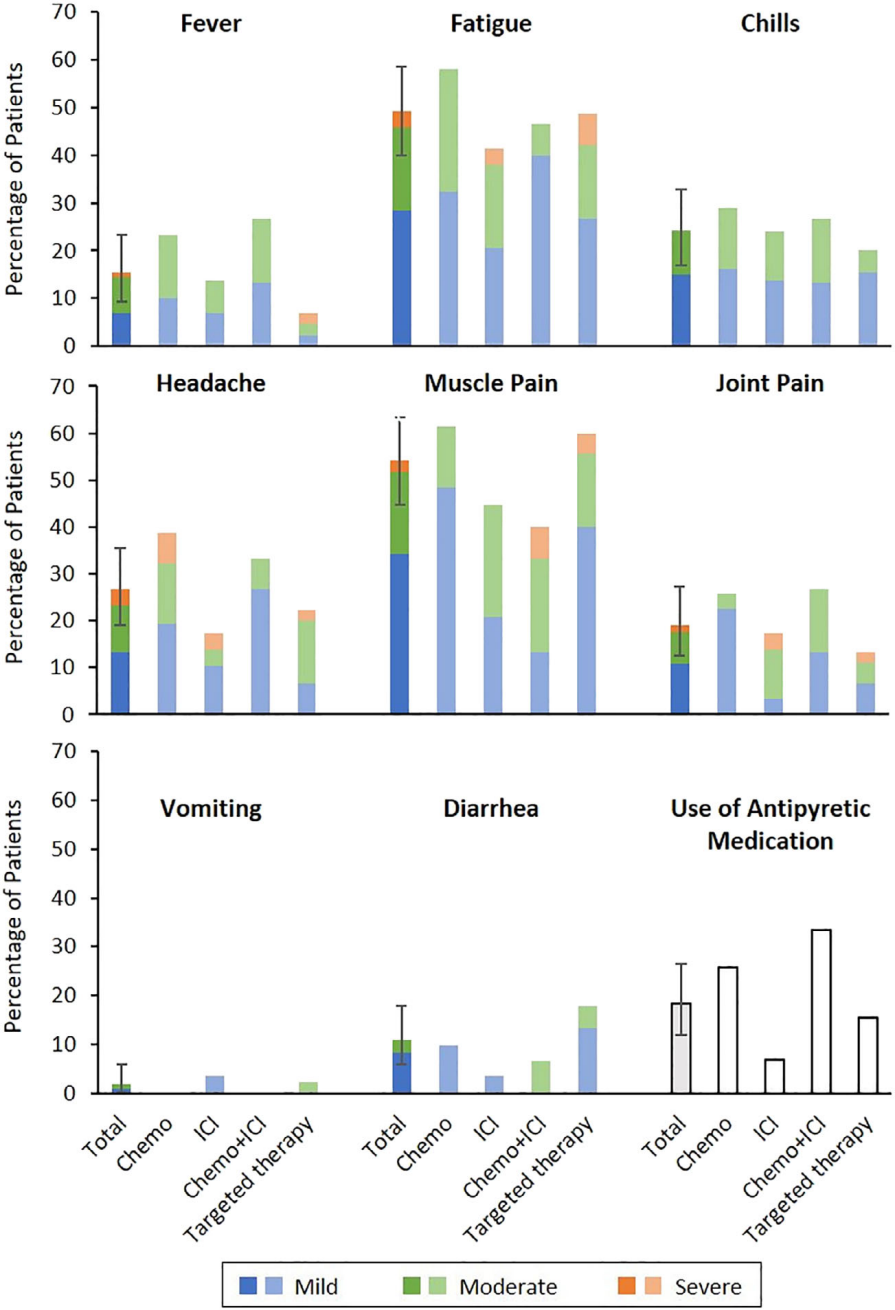
Systemic reactions reported after the second vaccine injection by treatment regimen. ICI, immune checkpoint inhibitor; Chemo, chemotherapy

**FIGURE 2:**
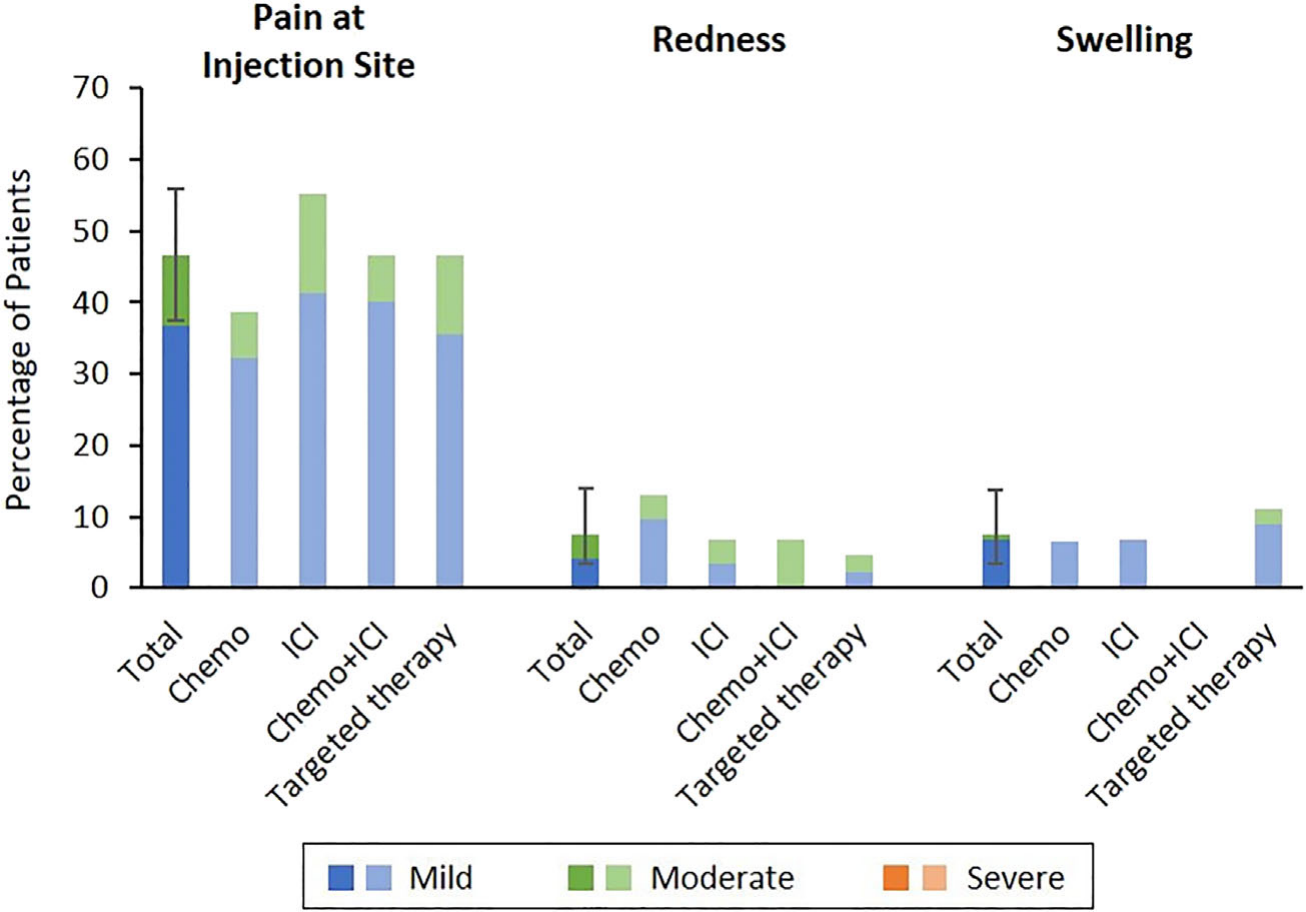
Local reactions reported after the second vaccine injection by treatment regimen. ICI, immune checkpoint inhibitor; Chemo, chemotherapy

Systemic reactions to the first and second injections are shown in Figure S1 and Figure 1, respectively. The frequency of fever >38°C after the first injection was 2.5%, and the frequency of fever >38°C after the second injection, the primary outcome, was 15.4% (95% confidence interval [CI]: 9.4%–23.2%). The frequency of fever for each treatment regimen is shown in Table 2. Fever after the second injection tended to be slightly more frequent with chemotherapy regimens and less frequent with targeted therapy. The most frequent systemic reactions after the second injection were myalgia (54.2%) and fatigue (49.2%), and there was no difference according to treatment regimens. The local reactions after the first and second inoculations are shown in Figure S2 and Figure 2, respectively. After the second injection, 46.7% of patients had pain at the injection site. However, there was no difference between the treatments. In total, no serious adverse events were observed in this study, and there were no cases in which the treatment schedule was postponed owing to adverse events of the vaccine.

**TABLE 2:** Frequency of fever after the second vaccine injection according to treatment regimen

Treatment regimen	Frequency	95% CI
Chemotherapy (*N* = 30^a^)	23.3%	9.9%–42.3%
ICI (*N* = 29)	13.8%	3.9%–31.7%
Chemotherapy plus ICI (*N* = 15)	26.7%	7.8%–55.1%
Targeted therapy (*N* = 43*)	7.0%	1.5%–19.1%

Abbreviations: CI, confidence interval; ICI, immune checkpoint inhibitor.

^**a**^ Three patients had missing values (one in the chemotherapy group and two in the targeted therapy group).

Patients receiving anticancer therapy, except targeted therapy, had the date of vaccine injection determined by their physician. There was little association between the period between anticancer drug administration and vaccination and adverse events, especially fever (Table S1). In addition, medications such as steroids and antipyretics, as well as adverse events such as fever had negligible effect on the results (Table S2). Univariate and multivariate analyses were performed to investigate the effects of fever. The frequency of fever was significantly higher in men than in women (adjusted odds ratio: 8.87; 95% CI: 1.25–62.8; *p* = 0.029). There was no difference in the frequency of fever between patients older and younger than 75 years (adjusted odds ratio: 1.73; 95% CI: 0.55–4.47; *p* = 0.350) (Table S3). Patients treated with cytotoxic anticancer drugs tended to have a high fever, and patients who received targeted therapy tended to have a lower frequency of fever, although the difference was not significant (Table 2, A.3).

## DISCUSSION

In the present study, the frequency of fever >38°C after the second injection, the primary outcome, was 15.4%. Compared with the findings in previous reports,^7^ the present findings suggested a higher risk in patients with lung cancer who are, or will be, receiving anticancer medicine than in healthy individuals. However, regarding other adverse events, many patients had muscle pain, although the degree was mild; in addition, other adverse events were similar to those reported in the BNT162b2 phase 3 trial,^7^ and the frequency of antipyretic use was also low (Figures 1 and S1). As a local reaction, pain was observed in many patients, but redness and swelling were less, and local pain tended to be less than the data reported in the BNT162b2 phase 3 trial (Figures 2 and S2).^7^ Although the frequency of fever after vaccination tends to be high, it is considered that the SARS‐CoV‐2 mRNA vaccine could be safely administered to patients with lung cancer.

Previous studies have reported that the side effects of the vaccine are low in patients undergoing cancer treatment.^13^ However, we obtained different results in our study. This may be because of differences in the methods of data collection or between races.

In our study, men tended to have fever more frequently than did women. A study by Menni et al. evaluated the safety and efficacy of the BNT162b2 and ChAdOx1 COVID‐19 vaccines in the UK and reported that women tended to have more adverse events than did men.^19^ The higher frequency of fever in men may be related to the fact that men had a higher frequency of a smoking history than,women, and smokers tend to have more fever than non‐smokers. Furthermore, because of the higher smoking rate among men than among women, the proportion of patients receiving targeted therapy was low, and the proportion of those receiving chemotherapy was high. Although adverse events tended to be more frequent in the chemotherapy group than in the nonchemotherapy group, the number of cases remains small at this time, and more data need to be collected to determine whether chemotherapy treatment will truly increase adverse events related to the vaccine. Patients who received chemotherapy tended to have more fever than those without chemotherapy, and none of them developed febrile neutropenia. The reason patients who received chemotherapy tended to have higher temperatures is unclear. Drug‐induced fever from anticancer drugs may have affected patients who were receiving chemotherapy. Currently, we are further accumulating cases, and we plan to verify our findings after the number of cases increases.

Our study has several limitations. First, our study included patients who had been using corticosteroids or antipyretic analgesics for treating lung cancer and complications prior to vaccination (Table S2). In these patients, preused drugs may have helped reduce adverse events. In addition, some patients had symptoms owing to lung cancer before vaccination, such as fatigue or any pain (Figures S3 and S4), and it is possible that the adverse events of the vaccine were overestimated in such patients. Second, our study could not determine whether the antibody titer increased; thus, the effectiveness of the vaccine could not be evaluated. Finally, since the main purpose of this report was to evaluate the short‐term safety of the COVID‐19 vaccine in those receiving lung cancer treatment, we could not examine its long‐term safety. Serious complications (stroke and myocardial infarction) and adverse events of immunotherapy have been the focus of recent attention^20^, ^21^ and will be examined in a number of cases during an observation period. At this time, our study is considered to be the result of ensuring the safety of vaccination in patients with lung cancer.

In conclusion, vaccine‐related adverse events tend to increase in patients with lung cancer undergoing cytotoxic chemotherapy. However, serious adverse events in the short‐term are comparable to those observed in healthy individuals. This cohort study provided data on the safety of using the mRNA vaccine for SARS‐CoV‐2 in patients with advanced lung cancer who are receiving anticancer therapies such as chemotherapy, ICIs, and targeted therapy.

## CONFLICT OF INTEREST

Toshio Kubo received lecture fees from Chugai pharmaceutical. Shoichi Kuyama received lecture fees from Chugai pharmaceutical. Yoshinobu Maeda received honoraria from Kyowa Kirin Co. Ltd., Bristol‐Myers Squibb Company, Chugai Pharma Co. Ltd., Pfizer Co. Ltd., Celgene Co. Ltd., Novartis Pharmaceutical Co. Ltd., and Takeda Pharmaceutical Co. Ltd., and research funding from Astellas Pharma Inc., Bristol‐Myers Squibb Company, Takeda Pharma Co. Ltd., Kyowa Kirin Co. Ltd., Nippon Shinyaku Co. Ltd and Chugai Pharma Co. Ltd. Katsuyuki Kiura received honoraria from MSD K.K., research funding from Pfizer Japan Inc., SHIONOGI & Co. Ltd., Boehringer Ingelheim Co. Ltd., Nippon Kayaku Co. Ltd., Taiho Pharmaceutical Co., Ltd., Ono Pharmaceutical Co. Ltd., MSD K.K., Chugai Pharmaceutical Co. Ltd., Bristol‐Myers Squibb K.K., Takeda Pharmaceutical Co. Ltd., and fees for consulting from Daiichi Sankyo Co. Ltd. The other authors declare that they have no known competing financial interests or personal relationships that could have appeared to influence the work reported in this study.

## Supporting information


**Figure S1**. Systemic reactions reported after the first vaccine injection by treatment regimen
ICI, immune checkpoint inhibitor; Chemo, chemotherapy

**Figure S2**. Local reactions reported after the first vaccine injection by treatment regimen
ICI, immune checkpoint inhibitor; Chemo, chemotherapy

**Figure S3**. Changes in systemic reactions before and after the first vaccine injection
Pre, preoperatively; Post, postoperatively

**Figure S4**. Changes in systemic reactions before and after the second vaccine injection
Pre, preoperatively; Post, postoperatively

**Table S1**. Duration of anticancer drugs administered before vaccine injection
**Table S2**. Frequency of medication use that affected the outcomes
**Table S3**. Odds ratio for fever of ≥38°C associated with the second vaccine injection
