# Supplementary figures and images for: Short‐term safety of an anti‐severe acute respiratory syndrome coronavirus 2 messenger RNA vaccine for patients with advanced lung cancer treated with anticancer drugs: A multicenter, prospective, observational study
Source: Thorac Cancer. 2021 Dec 28;13(3):453–9. doi: 10.1111/1759-7714.14281 (PMC8807248; doi:10.1111/1759-7714.14281)

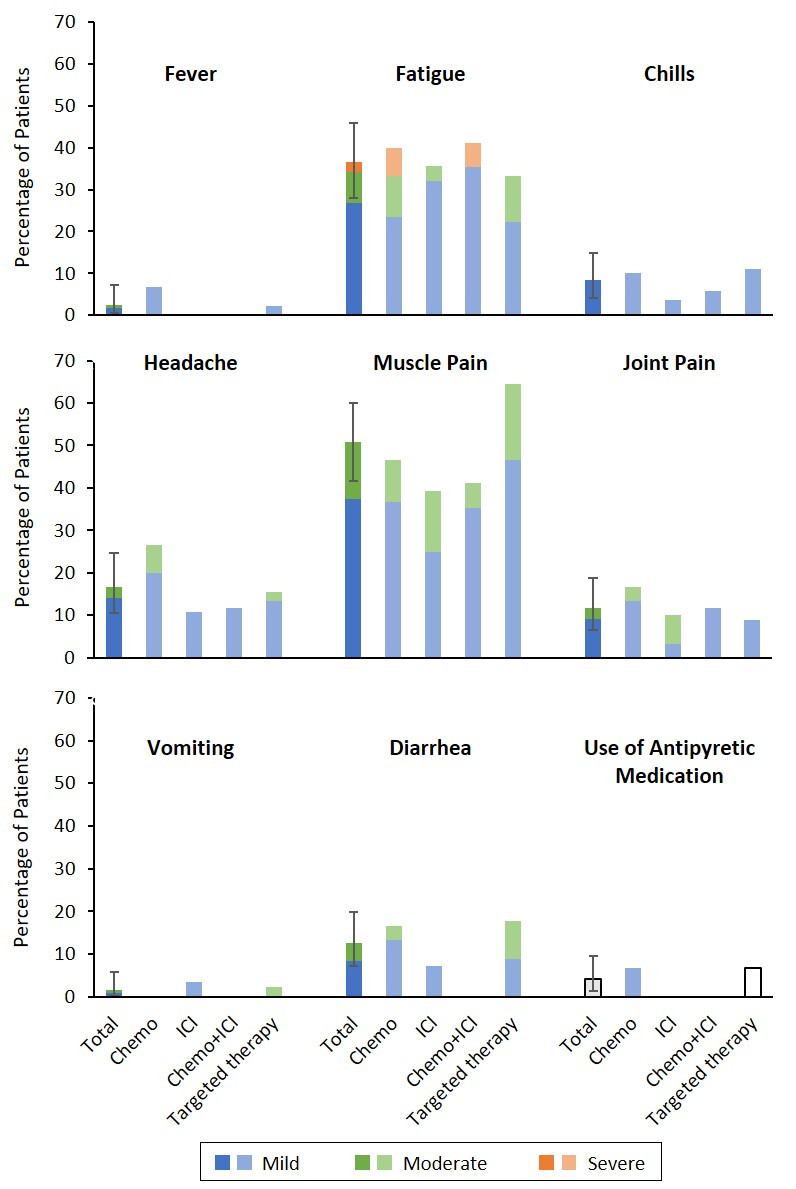

Supplement: Supplementary file 1 — Figure S1. Systemic reactions reported after the first vaccine injection by treatment regimen ICI, immune checkpoint inhibitor; Chemo, chemotherapy [file TCA-13-453-s002.jpg]

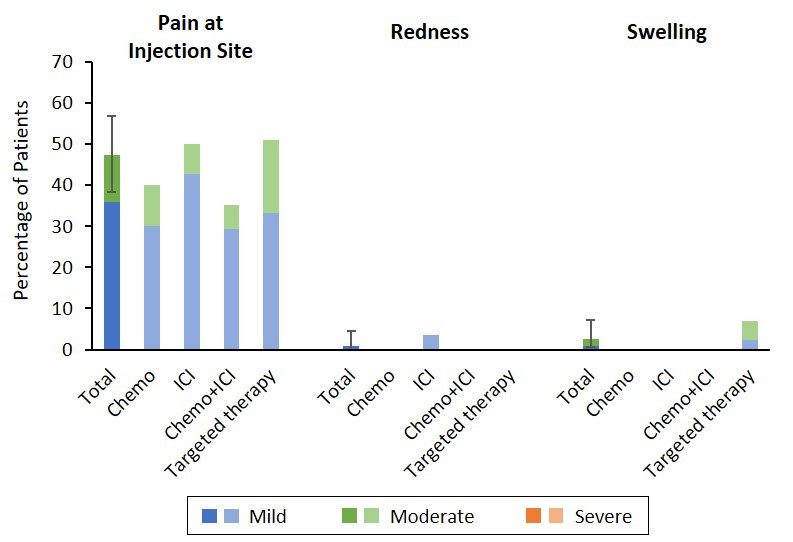

Supplement: Supplementary file 2 — Figure S2. Local reactions reported after the first vaccine injection by treatment regimen ICI, immune checkpoint inhibitor; Chemo, chemotherapy [file TCA-13-453-s005.jpg]

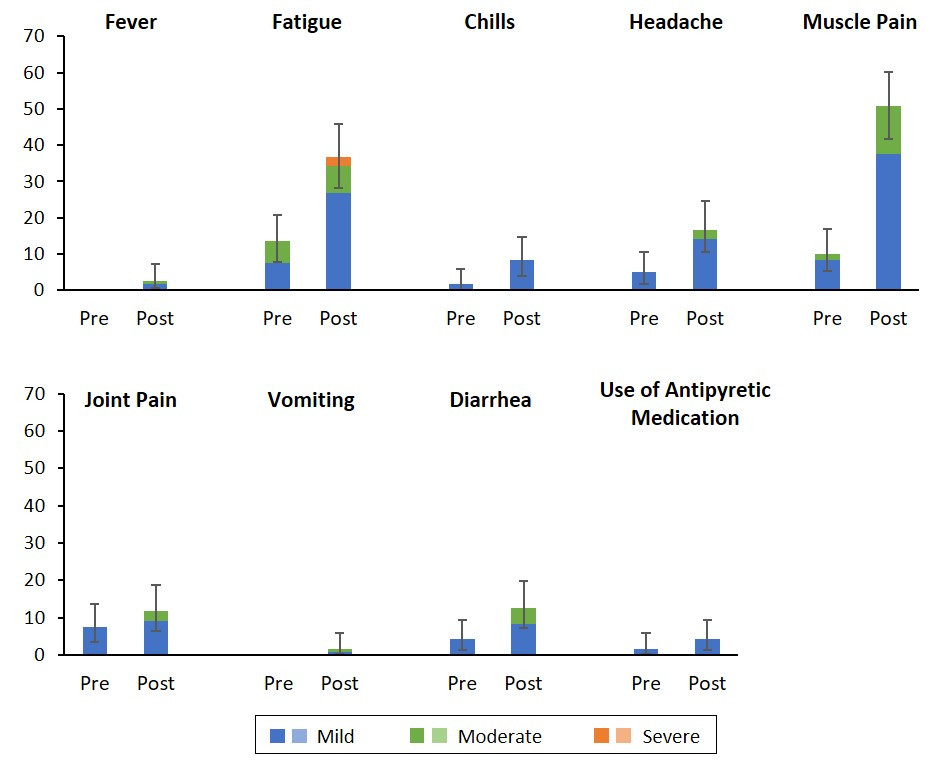

Supplement: Supplementary file 3 — Figure S3. Changes in systemic reactions before and after the first vaccine injection Pre, preoperatively; Post, postoperatively [file TCA-13-453-s003.jpg]

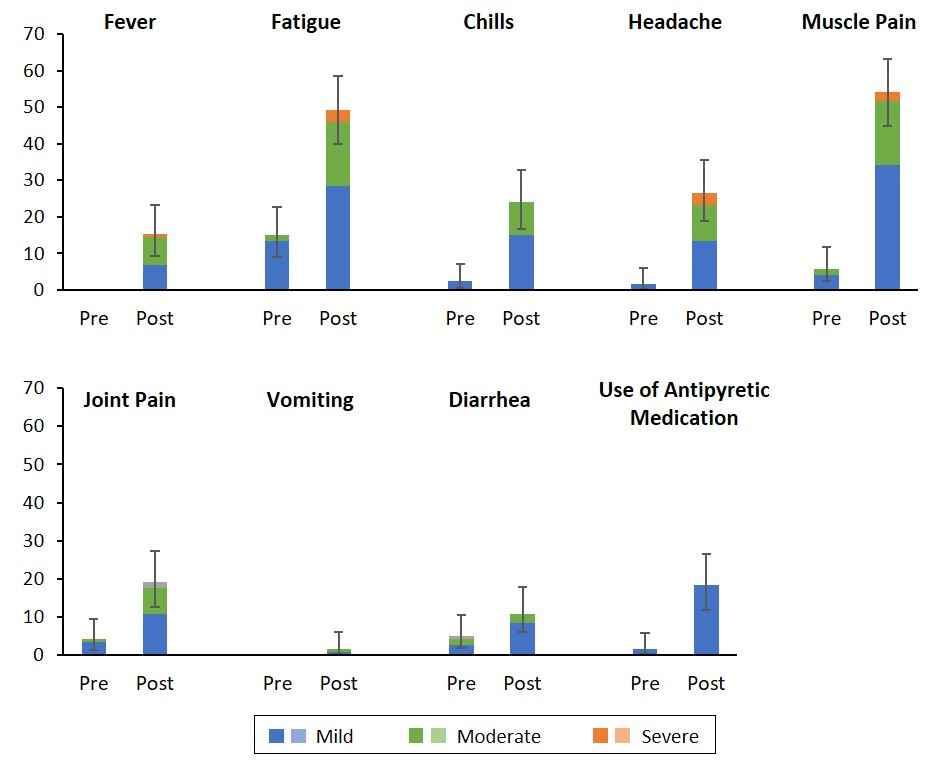

Supplement: Supplementary file 4 — Figure S4. Changes in systemic reactions before and after the second vaccine injection Pre, preoperatively; Post, postoperatively [file TCA-13-453-s004.jpg]
