# Supplementary material for: Short‐term safety of an anti‐severe acute respiratory syndrome coronavirus 2 messenger RNA vaccine for patients with advanced lung cancer treated with anticancer drugs: A multicenter, prospective, observational study
Source: Thorac Cancer. 2021 Dec 28;13(3):453–9. doi: 10.1111/1759-7714.14281 (PMC8807248; doi:10.1111/1759-7714.14281)
Supplement: Supplementary file 5 — Table S1. Duration of anticancer drugs administered before vaccine injection Table S2. Frequency of medication use that affected the outcomes Table S3. Odds ratio for fever of ≥38°C associated with the second vaccine injection [file TCA-13-453-s001.docx]

**Table S1. Duration of anticancer drugs administered before vaccine injection**

| Duration | No. (%) | Frequency of fever (95% CI) | p-value |
| --- | --- | --- | --- |
| First vaccine injection  Within 7 days  8–14 days  15–21 days  ≥22 days | 25 (33%)  20 (26%)  21 (28%)  10 (13%) | 4.0% (0.1%–20.4%)  0%  4.8% (0.1%–23.8%)  0% | 0.703 |
| Median [range] | 12 [1–105] |  |  |
| Second vaccine injection  Within 7 days  8–14 days  15–21 days  ≥22 days | 17 (22%)  31 (41%)  12 (16%)  16 (21%) | 17.6% (3.8%–43.4%)  16.7% (5.6%–34.7%)  25.0% (5.5%–57.2%)  25.0% (7.2%–52.4%) | 0.872 |
| Median [range] | 13 [0–126] |  |  |

Note: Targeted therapy did not have a withdrawal period.

Abbreviations: No., number; CI, confidence interval

**Table S2. Frequency of medication use that affected the outcomes**

| Medication | No. (%)* | Frequency of fever after the second vaccine injection | p-value |
| --- | --- | --- | --- |
| Steroids  Daily medication  Use as needed  None | 12 (10.3%)  4 (3.4%)  101 (86.3%) | 16.7% (2/12)  0% (0/4)  15.8% (16/101) | 0.731 |
| Antipyretic analgesics  Daily medication  Use as needed  None | 12 (10.3%)  9 (7.7%)  96 (82.1%) | 25.0% (3/12)  11.1% (1/9)  14.6% (14/96) | 0.599 |

*Three patients had missing values.

Abbreviation: No., number

**Table S3. Odds ratio for fever of ≥38°C associated with the second vaccine injection**

| Characteristic | Univariate analysis |  | Multivariate analysis | |
| --- | --- | --- | --- | --- |
|  | **Odds ratio (95% CI)** | **p-value** | **Odds ratio (95% CI)** | **p-value** |
| Institution | 1.15 (0.86–1.54) | 0.341 | 1.17 (0.84–1.62) | 0.341 |
| ≥75 years (vs. younger) | 1.28 (0.47–3.54) | 0.629 | 1.73 (0.55–5.47) | 0.350 |
| Men (vs. women) | 2.66 (1.09–14.70) | **0.037** | 8.87 (1.25–62.8) | **0.029** |
| ECOG-PS 1–2 (vs. ECOG-PS 0) | 0.68 (0.25–1.89) | 0.463 | 0.48 (0.15–1.55) | 0.219 |
| Smoker (vs. non-smoker) | 1.84 (0.61–5.56) | 0.281 | 0.26 (0.04–1.78) | 0.172 |
| Comorbid respiratory disease  (vs. non-comorbid respiratory disease) | 1.08 (0.35–3.32) | 0.893 | 0.66 (0.17–2.66) | 0.564 |
| Steroid use (vs. no steroid use) | 0.76 (0.16–3.67) | 0.731 | 1.02 (0.16 –6.46) | 0.986 |
| Antipyretic analgesic use  (vs. no antipyretic analgesic use ) | 1.38 (0.40–4.70) | 0.51 | 1.35 (0.35–5.17) | 0.660 |
| ICI use (vs. no ICI use) | 1.68 (0.61–4.60) | 0.316 | 0.95 (0.19–4.64) | 0.946 |
| Cytotoxic chemotherapy use  (vs. no cytotoxic chemotherapy use) | 2.29 (0.83–6.32) | 0.111 | 1.48 (0.31–6.94) | 0.620 |
| Targeted therapy use  (vs. no targeted therapy use) | 0.30 (0.80–1.09) | 0.066 | 0.31 (0.03–3.09) | 0.316 |

Abbreviations: CI, confidence interval; ICI, immune checkpoint inhibitor; vs., versus; ECOG-PS, Eastern Cooperative Oncology Group Performance Status
